# Management of Cerebrospinal Fluid Leak After Intrathecal Baclofen Pump Procedures in Adolescents With Cerebral Palsy: A Retrospective Study

**DOI:** 10.7759/cureus.61582

**Published:** 2024-06-03

**Authors:** B Randall Brenn, Joydeep Baidya, Dinesh K Choudhry

**Affiliations:** 1 Anesthesiology, Shriners Children's-Philadelphia, Philadelphia, USA; 2 Anesthesiology/Pediatric Anesthesiology, Vanderbilt University Medical Center, Nashville, USA; 3 Research, Cornell University, New York, USA

**Keywords:** post-dural puncture headache, epidural blood patch, spinal csf leak, spastic quadriplegia, treatment of cerebral palsy, spastic cerebral palsy, intrathecal pump therapy

## Abstract

Study objective: Epidural blood patches (EBPs) are frequently performed in children with cerebral palsy (CP) to manage post-dural puncture headache (PDPH) due to cerebrospinal fluid (CSF) leak after intrathecal baclofen pump (ITBP) placement or replacement procedures. The purpose of our study was to review the incidence and management of CSF leak following ITBP placement or replacement procedures in children with CP. The study was a retrospective review of 245 patients representing 310 surgical cases of baclofen pump insertion (n=141) or reinsertion (n=169) conducted at a 125-bed children’s hospital with prominent specialty orthopedics surgical cases.

Measurements: Demographic and clinical information was obtained from the anesthesia pain service database on all new ITBP placement and subsequent replacements over an eight-year period.

Main results: The overall incidence of CSF leak in our population was 16% (50 of 310) and 18% (25 of 141) with a new ITBP placement. Children with diplegia were associated with a threefold risk of developing CSF leak. Of patients who developed CSF leak (n=50), 68% (n=34) were successfully treated conservatively, while 32% (n=16) required EBPs. EBPs were successful in 87.5% (14 of 16) of patients at relieving PDPH on the first attempt.

Conclusions: CSF leak is a known problem after ITBP placement and replacement. Most patients were successfully treated with conservative management and EBPs were successful in patients failing conservative therapy. Diagnosing PDPH in non-verbal patients can be challenging.

## Introduction

Intrathecal drug delivery systems (IDDSs) have proven to be of benefit in patients with intractable pain and spasticity [1]. However, implantable pumps are not without complications. In addition to complications associated with the medications infused, there are surgical complications such as hematoma formation and surgical infections, equipment-related complications such as pump failure, catheter kinking or disconnection, and complications associated with cerebrospinal fluid (CSF) leak. CSF leaks can result in fluid accumulation in the epidural space or around the pump and can be associated with a post-dural puncture headache (PDPH) [2,3].

The Anesthesia Pain Service (APS) at our institution has performed epidural blood patches (EBPs) on several children with intractable spasticity following the placement or replacement of an intrathecal baclofen pump (ITBP), for either PDPH or for CSF leak not associated with documented headaches. The purpose of this study was to review our experience with CSF leaks after ITBP procedures and the occurrence of PDPH and evaluate the management leading to the use and efficacy of EBPs in these patients. 

## Materials and methods

All cases were from a 125-bed tertiary care, specialty orthopedic children’s hospital (Shriners Children's-Philadelphia, Philadelphia) in the mid-Atlantic region of the United States. Following Institutional Review Board approval, the records of all children who had ITBP placement or replacement procedures were reviewed. This included all those who had first-time catheter and pump placement and those who had replacement of a previously placed catheter or pump or both. At our institution, the orthopedic surgeons place all baclofen pumps for the treatment of spasticity. Data was collected retrospectively between the years 2008 and 2018 from our hospital's electronic medical record (Epic Systems, Verona WI). 

Patients were identified by CPT codes and data was extracted by searching a relational database constructed in a business intelligence platform (Qlikview, Radnor PA). This data was collated with patient data from the APS database (Microsoft Access, Microsoft Corporation, Bellevue WA) on patients who had EBP placed. Evidence of CSF leak included radiologic evidence or clinical evidence (PDPH reported by the patients or behavioral changes indicative of discomfort/headache related to posture change in non-verbal children). Obtained data were matched with pain service information on performed epidural blood patches.

Demographic information including patient name, medical record number, date of surgery, age, weight, orthopedic diagnosis, Gross Motor Function Classification System (GMFCS) level, procedures performed, procedure times, and recovery locations were recorded. Conservative management of PDPH included hydration, maintaining a flat posture, and caffeine therapy. In patients who had evidence of CSF leak, presence of headache, post-operative day of headache onset, other presenting symptoms, conservative therapies initiated, and EBP procedure details (day when EBP was performed, spinal level, and volume of blood instilled) were also recorded.

Statistical analysis

A univariate analysis was done to identify factors associated with CSF leak and EBP. Fisher’s exact test was used for nominal data and t-test for numeric data. Logistic regression was used to determine the odds ratios for factors associated with CSF leak and EBP. ANOVA was performed on each variable in order to evaluate their efficacy as accurate predictors in the logistic regression models. A p-value of 0.05 was considered significant. All analyses were implemented using R version 3.6.0 (https://www.r-project.org).

## Results

**Figure 1:**
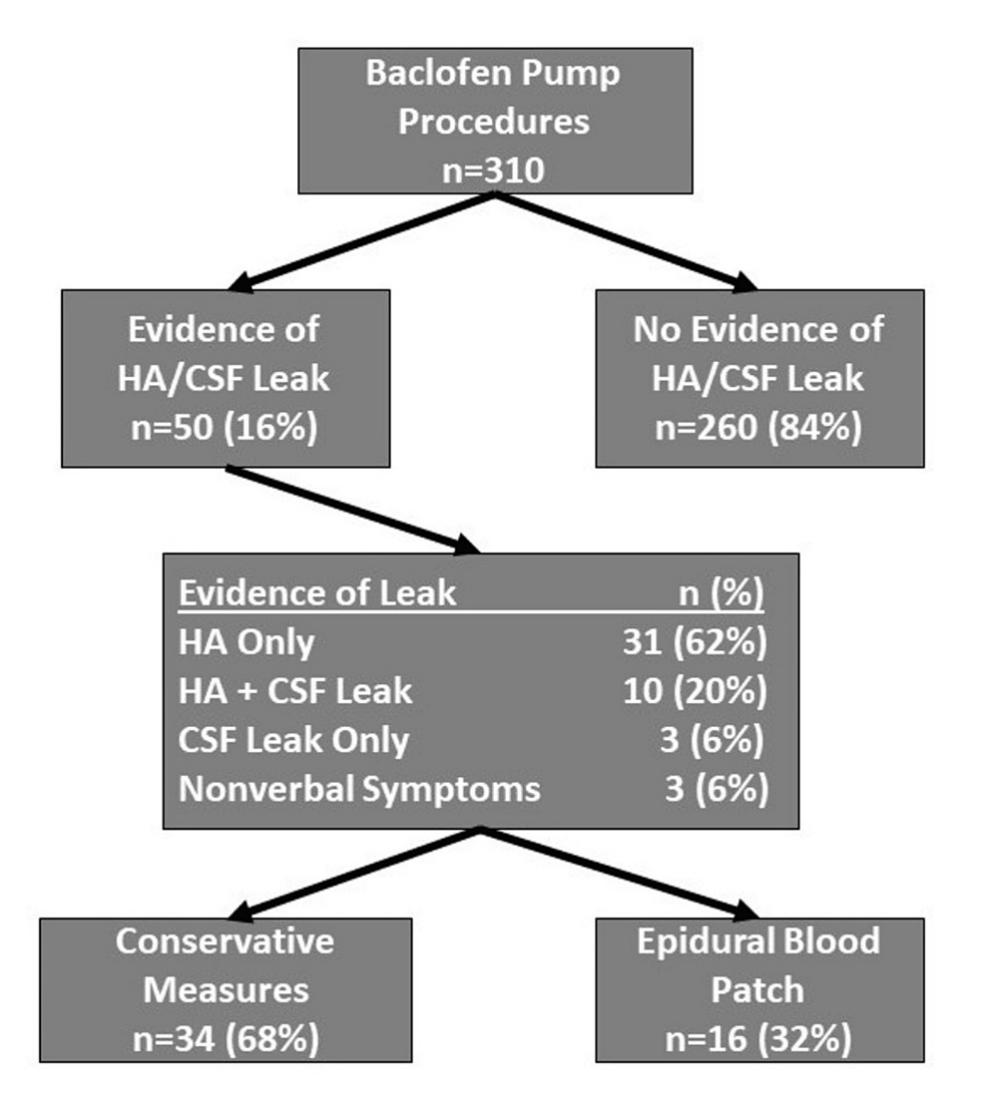
Baclofen pump procedures HA: Headache; CSF: Cerebrospinal Fluid

Data was collected on 310 surgical cases representing 245 patients. The overall scheme of the cases is demonstrated in Figure 1. Of 310 procedures, 50 (16%) had evidence of CSF leak. Of these 50, 41 (82%) had headaches (62%, n=31, had HA alone, 20%, n=10 had HA with radiographic evidence of CSF leak), and 3 (6%) had evidence of CSF leak without any evidence of headache. Of note, three patients (6%) who were non-verbal, had no evidence of CSF leak but had behavioral changes associated with change in posture that led the surgeons to suspect HA/CSF leak and request an EBP.

Demographic information is displayed in Table 1. In our population undergoing baclofen pump procedures, 264 (85%) of patients had spastic quadriplegia and 243 (78%) had a GMFCS level of V (non-ambulatory). The distribution of procedures and whether the baclofen pump procedure was combined with other procedures is also presented (Table 1). 

**Table 1:** Characteristics of the study population. GMFCS: Gross Motor Function Classification System; PACU: Post-anesthesia Care Unit; PICU: Pediatric Intensive Care Unit

Feature	N=310
Age - years, mean (SD)	13.90 (4.53)
Gender - n (%)	
Male	183 (59.03)
Female	127 (40.97)
Weight - kg, mean (SD)	38.12 (14.47)
Diagnosis - n (%)	
Quadriplegia	264 (85.16)
Diplegia	33 (10.65)
Dystonia	5 (1.61)
Athetoid	5 (1.61)
Hemiplegia	2 (0.65)
Spina Bifida	1 (0.32)
GMFCS Level - n (%)	
I	3 (0.97)
II	1 (0.32)
III	22 (7.10)
IV	41 (13.23)
V	243 (78.39)
Procedure - n (%)	
First Pump and Catheter	141 (45.48)
Replace Pump and Catheter	67 (21.61)
Replace Pump Only	85 (27.42)
Replace Catheter Only	13 (4.19)
Remove Pump and Catheter	4 (1.29)
Multiple (Combined) Procedures - n (%)	
Yes	271 (87.42)
No	39 (12.58)
Procedure Time - minutes, mean (SD)	
Total Surgical Time	197.23 (139.25)
Total PACU Time	114.33 (70.20)
Recovery Location - n (%)	
PACU	282 (90.97)
PICU	28 (9.03)

Table 2 displays the univariate analysis of variables associated with the development of a CSF leak. The weight, CP diagnosis, and GMFCS level variables were significantly associated with the development of a CSF leak. 

**Table 2:** Comparison of the variables associated with patients who did not and did develop CSF leak. ^1^: T-test;  ^2^: Fishers Test; GMFCS: Gross Motor Function Classification System; PACU: Post-anesthesia Care Unit;  PICU: Pediatric Intensive Care Unit;  CSF: Cerebrospinal Fluid

Variables	No CSF Leak	CSF Leak	p-value
	N=260 (84%)	N=50 (16%)	
Age-years, mean (SD)	13.76 (4.64)	14.64 (3.85)	0.158^1^
Gender-n (%)			
Male	154 (59.23)	29 (58.00)	0.877^2^
Female	106 (40.77)	21 (42.00)	
Weight-kg, mean (SD)	36.78 (13.72)	45.10 (16.33)	0.001^1^
Diagnosis-n (%)			
Quadriplegia	229 (88.08)	35 (70.00)	0.004^2^
Diplegia	23 (8.85)	10 (20.00)	
Other	8 (3.08)	5 (10.00)	
GMFCS Level - n (%)			
V	212 (81.54)	31 (62.00)	0.004^2^
Other	48 (18.46)	19 (38.00)	
Procedure-n (%)			
First Pump and Catheter	116 (44.62)	25 (50.00)	0.536^2^
Other	144 (55.38)	25 (50.00)	
Multiple (Combined) Procedures-n (%)			
Yes	226 (86.92)	45 (90.00)	0.648^2^
No	34 (13.08)	5 (10.00)	
Procedure Time-minutes, mean (SD)			
Total Surgical Time	196.90 (137.50)	198.94 (150.02)	0.929^1^
Total PACU Time	117.53 (70.96)	97.00 (63.94)	0.059^1^
Recovery Location-n (%)			
PACU	238 (91.54)	44 (88.00)	0.422^2^
PICU	22 (8.46)	6 (12.00)	

Logistic regression was used to tease out the characteristics of the factors that were deemed significantly associated with CSF leak, as demonstrated in Table 3. The patients’ weight, diagnoses, and GMFCS level were associated with CSF leak. Patients who weighed more (OR 1.036, 95% CI 1.016-1.057, p = .0004) were statistically associated with the development of a CSF leak. Compared to quadriplegic patients, the diplegic patients were associated with three times higher risk of CSF leak (OR 2.8, 95% CI 1.206-6.355, p=0.013). Also, patients with other diagnosis (which predominantly included dystonia and athetoid movements) were four times as likely to have CSF leak (OR 4.089, 95% CI 1.179-12.981, p=0.019). In addition, although most patients had a GMFCS level of V (non-ambulatory), those with lower GMFCS levels were observed to have a threefold higher risk of developing CSF leak (OR 2.707, 95% CI 1.396-5.168, p=0.003). Patients who weighed more, who were not wheelchair-bound, and who had non-quadriplegia (diplegia and other types) were more likely to need an EBP.

**Table 3:** Logistic regression for CSF leak. GMFCS: Gross Motor Function Classification System; PACU: Post-anesthesia Care Unit; PICU: Pediatric Intensive Care Unit; OR: Odds Ratio

Variables	OR	95% CI	OR p-value	ANOVA p-value
Age	1.045	0.977, 1.122	0.21	0.204
Gender	-	-	-	0.871
Male (reference)	1	-	-	-
Female	1.052	0.564, 1.938	0.871	-
Weight	1.036	1.016, 1.057	0.0004	0.0004
Diagnosis	-	-	-	0.009
Quadriplegia (reference)	1	-	-	-
Diplegia	2.845	1.206, 6.355	0.013	-
Other	4.089	1.179, 12.981	0.019	-
GMFCS Level	-	-	-	0.004
V (reference)	1	-	-	-
Other	2.707	1.396, 5.168	0.003	-
Procedure	-	-	-	0.485
First Pump and Catheter (reference)	1	-	-	-
Other	0.806	0.438, 1.480	0.484	-
Multiple (Combined) Procedures	-	-	-	0.538
Yes (reference)	1	-	-	-
No	0.739	0.243, 1.840	0.549	-
Procedure Time	-	-	-	-
Total Surgical Time	1	0.998, 1.002	0.924	0.925
Total PACU Time	0.994	0.987, 1.000	0.077	0.048
Recovery Location	-	-	-	0.44
PACU (reference)	1	-	-	-
PICU	1.475	0.519, 3.646	0.426	-

Table 4 demonstrates the characteristics of the patients who ultimately required EBPs. Sixteen patients, nine males and seven females, accounted for 17 EBP procedures. One patient had a repeat EBP, and another required a surgical procedure to apply fibrin glue to the CSF leak site. The average age was 14 years and ranged between 6 and 20 years. The average weight of the patients was 41.8 kg with a range of 22.4 kg to 80.9 kg. Seventy-five percent (12/16) of the patients were diagnosed with spastic quadriplegia and 12.5% (2/16) each were diagnosed with dystonia and diplegia. 

**Table 4:** Detailed table of the patients who received EBPs GMFCS: Gross Motor Function Classification System; PDPH: Post-dural Puncture Headache; CSF: Cerebrospinal Fluid; HA: Headache; EBP: Epidural Blood Patch; Quad: Quadriplegia; Dipl: Diplegia; Dyst: dystonia^1^; Mean value^ 2^: Median value.

Patient	Age (Yrs)	Weight (Kg)	Gender	Diagnosis	GMFCS	PDPH	CSF Leak	HA Day	EBP Day	Catheter Level	EBP Level	Volume Instilled
1	16	43.7	F	Quad	IV	No	Yes		18	L4-5	L5-S1	30
2	8	24.9	M	Quad	V	Yes	No	9	13	L4-5	L3-4	10
						Yes	No		25		L2-3	15
3	14	29.9	M	Quad	V	Yes	Yes	5	11	L2-3	L3-4	15
4	6	22.4	M	Quad	V	Yes	Yes	9	12	L2-3	L3-4	7.5
5	17	47.1	M	Dipl	III	Yes	Yes	3	91	L3-4	L3-4	15
6	16	56.4	F	Quad	V	Yes	No	2	7	L3-4	L5-S1	20
7	17	33.1	M	Quad	V	No	No		6	L2-3	L4-5	20
8	12	44	F	Quad	V	Yes	No	3	5	L2-3	L3-4	18
9	17	67.5	M	Dyst	II	Yes	No	3	6	L2-3	L4-5	20
10	13	39.6	F	Quad	V	No	Yes		2	L1-2	L1-2	20
11	8	25.9	M	Quad	V	No	No		10	L3-4	L4-5	10
12	17	30.5	F	Quad	V	No	No		10	L4-5	L2-3	15
13	11	25	F	Quad	V	No	Yes		14	L2-3	L4-5	15
14	19	80.9	M	Dyst	III	Yes	No	4	6	L3-4	L4-5	25
15	20	51.9	M	Dipl	II	Yes	No	2	4	L3-4	L4-5	30
16	20	63	F	Quad	IV	Yes	Yes	2	46	L3-4	L2-3	20
All	14^1^	41.8^1^							10^2^			18.7^1^

All EBPs were performed under fluoroscopic guidance, with a strict sterile technique at both the blood draw and the EBP sites. Over half of these, 10/17, (58.8%) were placed below the level of the intrathecal catheter insertion, 4/17 (23.5%) of EBPs above the level of the catheter, and 2/17 (11.7%) at the same level. 

Most patients developed symptoms in the first week after the procedure. The EBP was placed on the median post-surgical day 10, with the range being 2-91 days. One patient, who required a catheter replacement, received a blood patch on post-operative day 13 and then a repeat blood patch 25 days after the initial placement. The average volume of blood instilled was 18.7 ml, and the average volume instilled per kg was 0.4 ml/kg body weight. A second patient who also had a catheter only replacement received the EBP on day 6 but returned to the OR for fibrin glue application to stop a persistent CSF leak. The success rate of the first blood patch was 14/16 or 87.5%, whereas the remaining two patients needed either the second EBP or surgical intervention. 

## Discussion

Baclofen is a structural analog of gamma-aminobutyric acid and has been used as an intrathecal antispasticity agent since the early 1980s [4]. For the last two decades, intrathecal baclofen delivered by implantable pump systems has become commonplace and has improved the lives of many children with spasticity due to CP [5-7]. In children with CP, these systems are well tolerated with few complications [8,9]. Reports of CSF leaks range from 2 to 27% depending on the review [10,11]. 

Reports of PDPH after implantation with a baclofen pump in the literature are few. The rate of PDPH ranges from 0.6 to 8%, reported as a new headache in the post-operative period [9,10]. A recent report of PDPH associated with IDDSs showed an incidence of 22.9%5 and is typically treated conservatively. However, if the headaches are persistent, placement of an EBP is recommended [12-14]. In the report by Bendel et al., 20.5% of patients with PDPH after IDDS placement were treated with EBP when conservative management failed [15]. The placement of ITBP in this population has been reported to have few complications [9,16,17]. The incidence of CSF leak (2.2%) and CSF collection 3.3% was reported by Stempien et al. as a result of a survey of 115 institutions reporting on 936 pump placements [18]. However, smaller, single-institution series (23 and 25 patients) have reported CSF leaks of 16% [19] and 20% [10]. Our series of 310 procedures on 245 patients is consistent with a 16% rate. 

In our study, conservative measures were successful in treating two-thirds of the patients who experienced CSF leaks and the remaining third required EBP with 87% effectiveness. In addition, we observed that the risk of CSF leak was almost three times higher in more functional diplegic (and other) patients than quadriplegic patients. It is likely that less severely affected diplegic children were better able to be upright/ambulate and express their discomfort as compared to more profoundly delayed non-verbal, quadriplegic children where the diagnoses of CSF leak and headache could only be made by caregivers' observation of posture-related change in behavior from supine to elevated head position. The behaviors observed were agitation, moaning, crying, and excessive teeth grinding from lying down to sitting position. 

The incidence of PDPH (symptomatic CSF leak) has been reported by Neuman et al. [20] and Bendel et al. [15] as being as high as 23% in IDDS patients. However, their adult population consisted of predominantly chronic pain patients receiving intrathecal opioids and were distinctly different from our CP population. We included not only patients who could verbally express their headache, but also non-verbal patients who could not verbally express discomfort, and also patients with radiographic evidence of CSF leak. In Bendel’s larger review, 20.5% of patients with PDPH ultimately received EBP [15] whereas in our CP population, 34% required EBP to relieve the symptoms of CSF leak. 

The major limitation of our review is that it is retrospective where accuracy relies entirely on chart review and the potential for inconsistency in documentations exists. In addition, we suspect that the actual incidence of CSF leak may be higher in the quadriplegic population, as a significant number of these patients were non-verbal. In these children, the diagnosis of PDPH is based entirely on clinical suspicion based on noticing a change in their behavior associated with a change in posture. However, this is the largest review of the management of CSF leak after ITBP placement, and the use of EBP in the CP population.

## Conclusions

Baclofen delivered by ITBP, although a beneficial therapy for children with spasticity, is not without complications. Evidence of CSF leak manifested by PDPH or radiographic evidence of leak is successfully managed conservatively in two-thirds of the children, and the remainder could be successfully treated with EBPs. EBPs were successful at treating CSF leaks 87% of the time after one injection. Practitioners must maintain a high degree of suspicion in non-verbal patients who are unable to express PDPH but will likely benefit from EBPs.
